# The Neural Palette of Heme: Altered Heme Homeostasis Underlies Defective Neurotransmission, Increased Oxidative Stress, and Disease Pathogenesis

**DOI:** 10.3390/antiox13121441

**Published:** 2024-11-22

**Authors:** Adedamola Saidi Soladogun, Li Zhang

**Affiliations:** Department of Biological Sciences, School of Natural Sciences and Mathematics, University of Texas at Dallas, Richardson, TX 75080, USA; ass171930@utdallas.edu

**Keywords:** heme, neurobiology, neurotransmission, oxidative stress, mitochondria, neuroprotection, neurodegenerative diseases, synaptic plasticity, neural development, iron metabolism, neuroinflammation

## Abstract

Heme, a complex iron-containing molecule, is traditionally recognized for its pivotal role in oxygen transport and cellular respiration. However, emerging research has illuminated its multifaceted functions in the nervous system, extending beyond its canonical roles. This review delves into the diverse roles of heme in the nervous system, highlighting its involvement in neural development, neurotransmission, and neuroprotection. We discuss the molecular mechanisms by which heme modulates neuronal activity and synaptic plasticity, emphasizing its influence on ion channels and neurotransmitter receptors. Additionally, the review explores the potential neuroprotective properties of heme, examining its role in mitigating oxidative stress, including mitochondrial oxidative stress, and its implications in neurodegenerative diseases. Furthermore, we address the pathological consequences of heme dysregulation, linking it to conditions such as Alzheimer’s disease, Parkinson’s disease, and traumatic brain injuries. By providing a comprehensive overview of heme’s multifunctional roles in the nervous system, this review underscores its significance as a potential therapeutic target and diagnostic biomarker for various neurological disorders.

## 1. Introduction

Heme is traditionally recognized as the prosthetic group of hemoglobin and myoglobin; heme’s involvement in these proteins facilitates oxygen delivery to tissues and supports aerobic metabolism in mitochondria. The molecule’s significance extends into various biochemical pathways, crucial for maintaining cellular and systemic homeostasis [1].

Recent advances in neurobiology have uncovered that the roles of heme extend far beyond these traditional boundaries, particularly within the nervous system. This line of research reveals a multifaceted influence of heme on neural functions, from developmental processes to complex behavior modulations and neuroprotection [2,3]. The exploration of heme in the context of the nervous system is not merely an expansion of its known biological roles but a significant paradigm shift, suggesting that heme is as crucial for the brain’s molecular machinery as it is for the erythrocytes’ oxygen-carrying capacity.

The nervous system requires a meticulously controlled environment to function optimally, where even slight disturbances can lead to significant pathophysiological conditions. Heme has been identified as a critical regulator within this environment, influencing neuronal survival, growth, and repair mechanisms [4,5]. It interacts with various molecular pathways that govern neural health and disease, acting both as a signaling molecule and a modulator of cellular stress responses [6].

Neuroscientists are increasingly recognizing the importance of studying heme within the brain to better understand its comprehensive roles [5]. Such studies are critical not only for elucidating the fundamental aspects of neurobiology but also for paving the way toward novel therapeutic strategies against diverse neurological conditions [7,8]. Investigations into heme’s impact on neurodegenerative diseases, for instance, have demonstrated its potential involvement in the pathogenesis of disorders such as Alzheimer’s disease and Parkinson’s disease, where iron metabolism and oxidative stress play significant roles [5,8,9,10].

This growing body of research highlights the importance of considering heme not just as a minor component of neural function, but as a key player in the complex processes of the nervous system. By examining the molecular, cellular, and systemic roles of heme, this review aims to shed light on the crucial yet often overlooked functions of this molecule. It will explore how heme influences various aspects of neural activity and how its imbalance can contribute to disease, underscoring its potential as both a biomarker and a therapeutic target for neurodegenerative conditions.

## 2. Molecular Structure and General Functions of Heme

**Figure 1:**
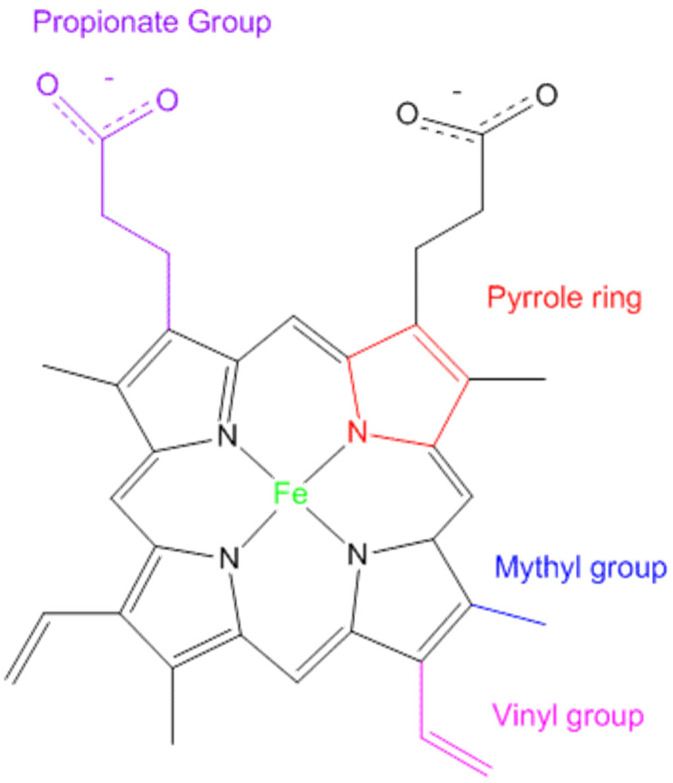
Molecular structure of heme. (Adapted from Wikipedia).

Heme is a complex molecule, integral to various biological processes due to its unique chemical structure [11,12]. Composed of an iron ion (Fe^2+^) centrally coordinated within a large heterocyclic organic ring known as porphyrin (Figure 1), heme’s structure allows it to participate in a multitude of critical functions. The porphyrin ring, characterized by four pyrrole subunits interconnected by methine bridges, creates a stable environment for the iron atom, which can bind various ligands [13,14,15]. This binding capability is fundamental to heme’s diverse roles in biology [16].

The iron atom at the core of the heme molecule can alternate between different oxidation states (Fe^2+^ and Fe^3+^), a property that enables heme to function as an electron carrier in oxidative phosphorylation and other metabolic pathways [17,18,19]. This alternation is essential for maintaining cellular homeostasis, as it allows for the efficient transfer of electrons, which is vital for energy production. Additionally, the planar, hydrophobic nature of the porphyrin ring allows it to insert seamlessly into protein matrices, stabilizing their structures and enhancing their functions [20,21].

One of the most prominent roles of heme is in cellular respiration, particularly within the mitochondrial electron transport chain [22,23]. Cytochromes, which are heme-containing proteins, are crucial in this process [24]. For instance, cytochrome c plays a key role in transferring electrons from complex III to complex IV, ultimately facilitating the reduction of oxygen to water [25,26]. This reduction is a pivotal step in the generation of ATP, the cell’s primary energy currency, underscoring heme’s central role in cellular energy metabolism [27].

In oxygen transport, heme’s significance is exemplified by its presence in hemoglobin, the oxygen-carrying protein in red blood cells. Each hemoglobin molecule contains four heme groups, each capable of binding one molecule of oxygen [28,29]. This reversible binding enables hemoglobin to efficiently capture oxygen in the lungs and release it in tissues where it is needed for metabolic processes [30]. The dynamic nature of this binding and release is facilitated by conformational changes in the hemoglobin molecule, a phenomenon known as cooperative binding [31,32], where the binding of one oxygen molecule increases the affinity of the remaining heme groups for oxygen [33,34].

Beyond its roles in electron transport and oxygen carriage, heme is involved in various enzymatic functions. Heme acts as a cofactor in catalase, an enzyme that decomposes hydrogen peroxide into water and oxygen, thereby protecting cells from oxidative damage [35,36]. Additionally, heme-containing enzymes such as peroxidases and cytochrome P450s are integral to the metabolism of a wide array of substrates, including both xenobiotics and endogenous compounds [37]. These enzymes are critical for detoxifying harmful substances and synthesizing essential biomolecules, underscoring heme’s versatile role in cellular metabolism [38,39].

Furthermore, heme’s regulatory functions extend to gene expression and signal transduction [4,40]. Heme can influence the activity of transcription factors and other regulatory proteins, modulating the expression of genes involved in various physiological processes [41,42,43,44]. For example, heme regulates the activity of heme-responsive transcription factors, which control the expression of genes related to heme synthesis and degradation. This regulatory role is crucial for maintaining heme homeostasis within cells, preventing both heme deficiency and toxicity [45,46].

In summary, heme’s intricate chemical structure and multifaceted functions underscore its importance in biology. Its ability to bind and transport oxygen, facilitate electron transfer, and regulate enzymatic activity makes it indispensable for cellular respiration, oxygen transport, and metabolic processes. Additionally, heme’s role in gene regulation highlights its broader impact on cellular function and homeostasis, establishing it as a vital molecule in both health and disease [47,48,49].

## 3. Molecular Details Involving Heme–Regulatory Protein Interactions

**Figure 2:**
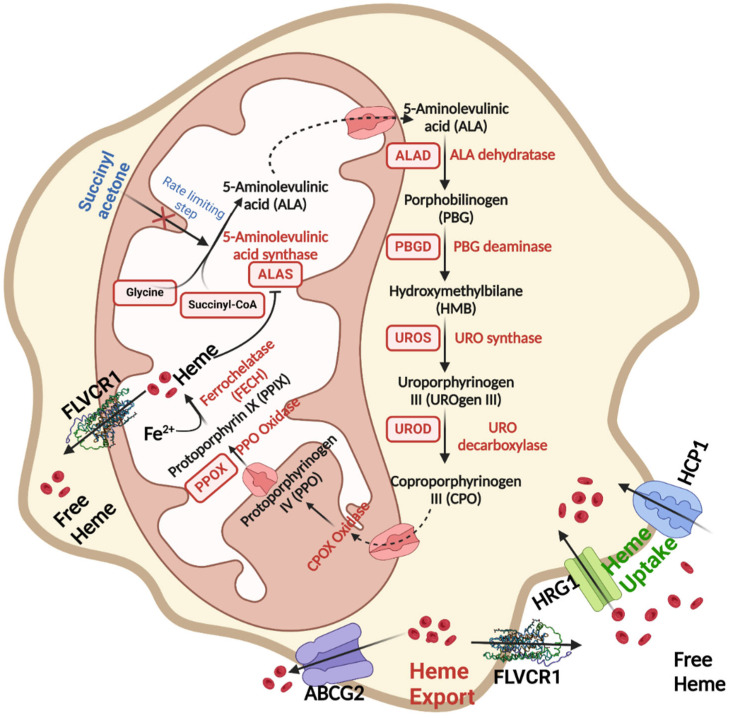
Heme biosynthetic and transport pathway. **FLVCR1**-Feline Leukemia Virus Subgroup C Receptor 1. **HCP1**-Heme Carrier Protein 1. **HRG1**-Heme Responsive Gene 1 Protein Homolog. **ABCG2**-ATP-Binding Cassette Subfamily G Member 2. Created in BioRender. Soladogun, A. (2024) www.BioRender.com/a43u711 (accessed on 16 October 2024).

**Figure 3:**
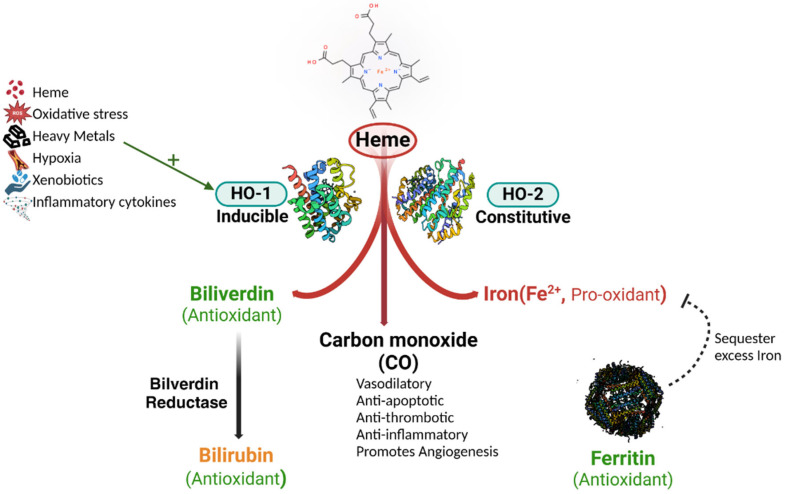
The heme degradation pathway modulates several cellular processes. Created in BioRender. Soladogun, A. (2024). www.BioRender.com/d68u922 (accessed on 16 October 2024).

Heme’s interactions with various regulatory proteins play a pivotal role in modulating its function, transport, and degradation (Figure 2 and Figure 3). These interactions ensure intracellular heme homeostasis, a critical balance that, when disrupted, can lead to pathophysiological conditions ranging from increased oxidative stress to neurodegenerative disorders [5]. In the nervous system, heme–regulatory protein interactions influence cellular respiration, neurotransmitter synthesis, and redox signaling, making them integral to neural health and disease. Analyzing these interactions at a molecular level provides insights into therapeutic targets for neurological disorders where heme dysregulation is a hallmark feature.

### 3.1. Heme and Heme Oxygenase (HO) Interaction

Heme oxygenase (HO) is the primary enzyme responsible for heme catabolism, converting heme into biliverdin, carbon monoxide (CO), and free iron (Figure 3). There are two major isoforms, HO-1 and HO-2, with distinct roles in cellular physiology. HO-1 is inducible and expressed in response to oxidative stress, while HO-2 is constitutively expressed and found predominantly in the brain and testes [50].

#### 3.1.1. Mechanism of Heme Degradation

The catalytic activity of HO involves the cleavage of the α-methene bridge of heme, which requires molecular oxygen and NADPH-cytochrome P450 reductase as reducing equivalents [51,52]. The resultant byproducts have diverse biological functions: biliverdin is further reduced to bilirubin, a potent antioxidant, by biliverdin reductase [53]; CO acts as a signaling molecule that modulates vasodilation and anti-inflammatory responses [54,55]; and free iron is sequestered by ferritin to prevent iron-mediated oxidative damage [56,57].

#### 3.1.2. Regulation of HO Activity

HO-1 expression is regulated by nuclear factor erythroid 2–related factor 2 (Nrf2), which binds to antioxidant response elements (AREs) in the HO-1 promoter region upon oxidative stress [58,59]. Bach1, a transcriptional repressor, inhibits HO-1 expression under basal conditions by competing with Nrf2 for ARE binding. Heme itself can disrupt Bach1’s DNA-binding ability, thereby promoting HO-1 transcription [40,60,61].

#### 3.1.3. Physiological Significance of HO Byproducts

Bilirubin has been shown to protect neurons from oxidative damage by scavenging superoxide [62]. CO exerts neuroprotective effects by activating soluble guanylate cyclase and modulating mitochondrial function [63,64]. Dysregulation of HO activity has been observed in various neurodegenerative conditions, such as Alzheimer’s disease and Parkinson’s disease, where altered HO-1 expression correlates with increased oxidative damage and neuroinflammation [65,66].

### 3.2. Heme and Nitric Oxide Synthase (NOS) Interaction

Nitric oxide synthase (NOS) enzymes are responsible for the production of nitric oxide (NO), a signaling molecule essential for vascular tone, neurotransmission, and immune responses. Heme is a critical cofactor for all three isoforms of NOS—neuronal NOS (nNOS), endothelial NOS (eNOS), and inducible NOS (iNOS). These enzymes catalyze the conversion of L-arginine to NO, a process heavily dependent on the availability of heme. [67,68].

#### 3.2.1. NOS Isoforms and Heme Binding

The catalytic activity of NOS enzymes requires heme to bind oxygen and facilitate the synthesis of NO from L-arginine. In the heme-containing oxygenase domain of NOS, heme enables the activation of molecular oxygen, which is necessary for the subsequent oxidation of L-arginine to citrulline and NO [69]. Heme is also essential for electron transfer in the catalytic cycle, and alterations in heme availability can lead to impaired NOS function [70,71].

#### 3.2.2. Role of Heme in NOS Regulation

Heme plays a critical role in the regulation of NOS activity. For instance, in the endothelial system, eNOS-derived NO is crucial for maintaining vascular homeostasis. Reduced bioavailability of heme has been shown to decrease NO production, contributing to endothelial dysfunction, hypertension, and atherosclerosis [72]. In the nervous system, nNOS-derived NO regulates synaptic plasticity and neurotransmitter release [73,74], and dysregulated NO signaling has been implicated in neurodegenerative diseases such as Parkinson’s and Alzheimer’s [75,76].

#### 3.2.3. Pathophysiological Implications

Dysregulation of NOS activity, particularly due to insufficient heme availability, can have deleterious effects. In ischemic conditions, for instance, increased iNOS activity can exacerbate neuronal damage by producing excessive amounts of NO, leading to the formation of peroxynitrite, a reactive nitrogen species that induces oxidative and nitrosative stress [77]. This pathway is particularly relevant in conditions such as stroke, where heightened NO production contributes to neurodegeneration [78].

### 3.3. Heme and Cytochrome P450 Interaction

Cytochrome P450 (CYP) enzymes are heme-dependent monooxygenases that play a critical role in the metabolism of a wide range of substrates, including drugs, xenobiotics, and endogenous compounds. These enzymes are integral to detoxification processes and the biosynthesis of important signaling molecules, such as steroid hormones.

#### 3.3.1. Catalytic Function of Cytochrome P450

The heme prosthetic group within CYP enzymes binds oxygen and facilitates its activation, a crucial step in the oxidative metabolism of substrates. The catalytic cycle of CYP begins with the binding of a substrate to the enzyme, followed by the transfer of electrons from NADPH via cytochrome P450 reductase, which reduces the heme iron from the ferric (Fe^3+^) to the ferrous (Fe^2+^) state. Oxygen then binds to the reduced heme, forming a dioxygen complex that enables substrate hydroxylation [79,80].

#### 3.3.2. Regulation of CYP Activity by Heme Availability

Heme availability is a key regulatory factor for CYP enzyme activity. Heme deficiency has been shown to impair CYP function, leading to reduced capacity for xenobiotic metabolism and increased susceptibility to oxidative stress [81,82]. On the other hand, excess heme production can drive the overexpression of CYP enzymes, which can enhance the generation of reactive oxygen species (ROS) and promote oxidative damage [83].

#### 3.3.3. Neurodegenerative Implications of CYP Dysregulation

Several CYP isoforms are expressed in the brain, where they are involved in the metabolism of neuroactive compounds, including neurotransmitters and neurosteroids [84,85,86]. Dysregulation of CYP enzymes has been implicated in the pathophysiology of neurodegenerative diseases. For example, the impaired CYP-mediated metabolism of cholesterol and other lipids has been linked to the pathogenesis of neurodegenerative diseases [87,88].

### 3.4. Other Heme–Regulatory Protein Interactions

Heme’s regulatory role extends beyond its involvement with key metabolic enzymes, influencing several other proteins that regulate gene expression, iron homeostasis, and cellular stress responses. These interactions underscore heme’s importance as a signaling molecule in various physiological and pathological processes.

#### 3.4.1. Heme and Bach1

Bach1 is a transcriptional repressor that controls the expression of genes involved in the antioxidant response, most notably heme oxygenase-1 (HO-1) [61]. Under normal conditions, Bach1 binds to antioxidant response elements (AREs) in the promoter regions of target genes, inhibiting their transcription. Heme can bind directly to Bach1, leading to its dissociation from the ARE and allowing Nrf2 to induce the expression of HO-1, which provides cytoprotection against oxidative stress [89]. This mechanism is critical for cellular adaptation to stress and has been implicated in neuroprotection in models of neurodegenerative diseases [90,91].

#### 3.4.2. Heme and Iron Regulatory Proteins (IRPs)

Heme plays an important role in regulating intracellular iron levels through its interaction with iron regulatory proteins (IRPs) [92]. IRPs modulate the translation of mRNAs encoding ferritin (an iron storage protein) and transferrin receptor (an iron importer), thus maintaining cellular iron balance. Heme inhibits the RNA-binding activity of IRP1, promoting ferritin synthesis and reducing iron import, thereby preventing iron overload and associated oxidative damage [93,94,95]. Disruption of this regulatory mechanism can result in abnormal iron accumulation, a feature observed in neurodegenerative diseases like Alzheimer’s and Parkinson’s as well as neurodegeneration with brain iron (NBIA) diseases [96,97].

#### 3.4.3. Heme and Heat Shock Proteins (HSPs)

Heme interacts with heat shock proteins (HSPs), which are involved in protein folding, stabilization, and the cellular stress response. Heme binding to HSP32, also known as HO-1, promotes its stability and function, enabling cells to cope with oxidative stress and prevent protein misfolding. The upregulation of HSP32 in response to increased heme levels has been shown to protect neurons from oxidative damage in models of neurodegeneration [98].

## 4. Heme in Neural Development

Neuronal differentiation involves the transformation of precursor cells into mature neurons, a process crucial for brain development and function. Heme is indispensable for neuronal survival due to its involvement in numerous cellular processes that ensure the proper functioning and resilience of neurons. Studies have shown that heme deficiency leads to neuronal death [99,100]. Heme supports the metabolic and antioxidative needs of neurons, particularly through its regulation of the nuclear factor erythroid 2–related factor 2 (Nrf2) [101]. Heme indirectly activates Nrf2 by binding to Bach1, a transcriptional repressor that normally inhibits Nrf2-targeted genes. The binding of heme to Bach1 promotes its nuclear export, allowing Nrf2 to accumulate and translocate into the nucleus, where it binds to antioxidant response elements (AREs) and upregulates genes essential for antioxidant defense and neuronal resilience [61,102]. This activation supports neuron survival during differentiation by enhancing cellular resilience to oxidative stress [103,104].

Additionally, heme oxygenase-1 (HO-1), an enzyme that degrades heme, produces byproducts such as biliverdin, carbon monoxide, and free iron [105,106]. These byproducts have been shown to possess neuroprotective properties, promoting the survival and maturation of neurons. For example, studies have demonstrated that HO-1 overexpression and upregulation during brain injury facilitate neuronal recovery and maturation by mitigating oxidative stress [107,108].

### Influence on Axonal Guidance and Synaptic Formation

Heme also plays a pivotal role in axonal guidance, the process by which neurons extend axons to reach their correct targets, and in synaptic formation, both of which are crucial for establishing functional neural circuits. Heme regulates the activity of several proteins and enzymes involved in these processes, particularly through its role as a cofactor for cytochrome P450 enzymes. These heme-dependent enzymes metabolize various signaling molecules essential for neural development, including retinoic acid, a molecule that influences the patterning and growth of axons [109,110,111]. Cytochrome P450 enzymes hydroxylate retinoic acid, converting it to active metabolites that interact with nuclear retinoic acid receptors (RARs) in neurons, thereby driving gene transcription pathways that guide axonal growth and establish spatial organization.

Heme indirectly supports these functions by enabling cytochrome P450 enzymes to generate necessary signaling molecules, such as retinoic acid, that guide axonal development and synaptic formation. Without adequate heme, cytochrome P450 activity diminishes, leading to disrupted retinoic acid metabolism and impairing axonal guidance. This can result in aberrant neural circuit formation and reduced synaptic plasticity, impacting learning and memory [112]. The influence of heme on these enzymatic processes highlights its indirect yet critical role in ensuring proper axonal targeting and synaptic connectivity, which are fundamental to cognitive function.

Moreover, heme regulates synaptic plasticity, the ability of synapses to strengthen or weaken over time, which is fundamental for learning and memory. Heme’s influence on synaptic plasticity is partly mediated through its role in the synthesis of the neurotransmitter, nitric oxide (NO). Heme-containing nitric oxide synthase (NOS) enzymes produce NO [113], which modulates synaptic strength and plasticity. Heme dysregulation can cause alterations in NO signaling which impact synaptic formation and function, highlighting heme’s critical role in neural connectivity [75,114]. For instance, NO produced by NOS plays a crucial role in synaptic plasticity by modulating the strength and efficacy of synaptic transmission, a process essential for cognitive functions such as learning and memory [74,115]. Additionally, NO signaling pathways are implicated in various neuroprotective mechanisms, which help maintain neuronal health and function [116,117,118,119].

In summary, heme’s intricate chemical structure and multifaceted functions underscore its importance in neural development. Its ability to influence neuronal differentiation and maturation, facilitate axonal guidance, and regulate synaptic formation makes it indispensable for the proper formation and function of the nervous system.

## 5. Heme and Neurotransmission

Heme is a critical player in the regulation of neurotransmission, which is fundamental to the proper functioning of the nervous system. Neurotransmission is the process by which neurons communicate with each other and with other cells, and it involves the release, reception, and modulation of chemical signals known as neurotransmitters. Heme influences this process through multiple mechanisms, including the modulation of ion channels and the synthesis of neurotransmitters.

A notable example of heme’s influence on neurotransmission is its modulation of potassium channels, which are vital for maintaining neuronal excitability and signal transmission. Potassium channels help maintain the resting membrane potential and regulate action potentials in neurons [120,121,122]. Heme can bind to these channels, altering their activity and thereby impacting neuronal signaling. Studies have shown that changes in heme levels can lead to significant alterations in the function of specific potassium channels, affecting the overall neuronal communication and excitability [123,124,125]. This modulation is critical for ensuring proper neuronal function and maintaining effective neural circuits [126].

Continuing this theme, heme also modulates N-methyl-D-aspartate (NMDA) receptors, which are crucial for synaptic plasticity and memory function [127,128]. NMDA receptors are ligand-gated ion channels involved in excitatory neurotransmission in the brain. NMDA receptors are activated when glutamate binds to them in the presence of membrane depolarization, allowing Ca^2+^ ions to enter the neuron. Heme deficiency has been linked to reduced NMDA receptor expression and impaired synaptic transmission. This effect is mediated through the inhibition of the extracellular signal-regulated kinase (ERK) pathway, which is crucial for NMDA receptor function and neuronal survival [100]. Research has shown that depleted heme levels lead to reduced NMDA receptor expression, thereby affecting synaptic transmission and plasticity [129]. Moreover, bilirubin, a product of heme degradation, has been shown to activate NMDA receptors in the developing brain [130].

In addition to modulating ion channels, heme is indirectly involved in the synthesis of several key neurotransmitters. Iron, which can be obtained from heme degradation, acts as a cofactor for enzymes such as tryptophan hydroxylase and tyrosine hydroxylase, which are critical for the synthesis of serotonin and dopamine, respectively [131,132]. Tryptophan hydroxylase catalyzes the hydroxylation of tryptophan, the rate-limiting step in serotonin synthesis, while tyrosine hydroxylase catalyzes the hydroxylation of tyrosine, the first step in dopamine synthesis. Iron molecule enables the transfer of oxygen in these reactions, producing serotonin and dopamine precursors essential for maintaining mood, cognitive functions, and reward pathways. Furthermore, heme-bound amyloid-beta (Aβ) can catalyze the oxidative degradation of serotonin, producing neurotoxic compounds. This degradation process is most effective at physiological pH and involves key residues such as Arg5 and Tyr10 in the heme–Aβ complex [133]. This highlights the role of heme in both the synthesis and regulation of neurotransmitters, emphasizing its importance in neurological health and function.

Lastly, heme influences nitric oxide (NO) signaling through its role as a cofactor for nitric oxide synthase (NOS), which produces NO, a gaseous neurotransmitter with diverse functions in the nervous system, including modulation of synaptic plasticity and memory formation. NO acts as a retrograde signaling molecule, diffusing back to presynaptic neurons to modulate neurotransmitter release [134,135,136]. Heme within NOS enzymes facilitates the conversion of L-arginine to NO by transferring the electrons necessary for the reaction [137]. NO interacts with soluble guanylate cyclase (sGC) in target cells, leading to cyclic GMP (cGMP) production and downstream signaling events that modulate synaptic strength and plasticity [138]. Alterations in NO signaling, due to heme dysregulation, can impair these signaling pathways, affecting synaptic formation and function and highlighting heme’s role in maintaining neural circuit plasticity and adaptability [74,75].

## 6. Antioxidant and Neuroprotective Functions of Heme

Heme plays a crucial role in the nervous system both through its involvement in cellular respiration and neurotransmission and also by serving as a potent antioxidant and contributing to cellular defense mechanisms against oxidative stress. The neuroprotective properties of heme are essential for maintaining neuronal health and function, especially in the context of neurodegenerative diseases and other conditions characterized by increased oxidative damage.

### 6.1. Heme as an Antioxidant in the Nervous System

One of the primary neuroprotective roles of heme is its function as an antioxidant. Antioxidants are molecules that can prevent or slow the damage to cells caused by free radicals, unstable molecules that can cause oxidative stress [139]. Heme exerts its antioxidant effects through its role in various heme-containing enzymes, such as catalase and peroxidases, which help neutralize reactive oxygen species (ROS).

Catalase is a critical enzyme in the detoxification of hydrogen peroxide (H_2_O_2_), a common ROS [140]. Heme serves as a cofactor in catalase, enabling the conversion of hydrogen peroxide into water and oxygen, thereby mitigating potential cellular damage. Similarly, peroxidases, another group of heme-containing enzymes, contribute to the breakdown of peroxides, further reducing oxidative stress within neural tissues [141]. By maintaining the balance of ROS, these enzymes help protect neurons from oxidative damage, which is crucial for preserving cognitive functions and preventing neurodegenerative diseases [142,143].

### 6.2. Role in Cellular Defense Mechanisms Against Oxidative Stress and Inflammation

**Figure 4:**
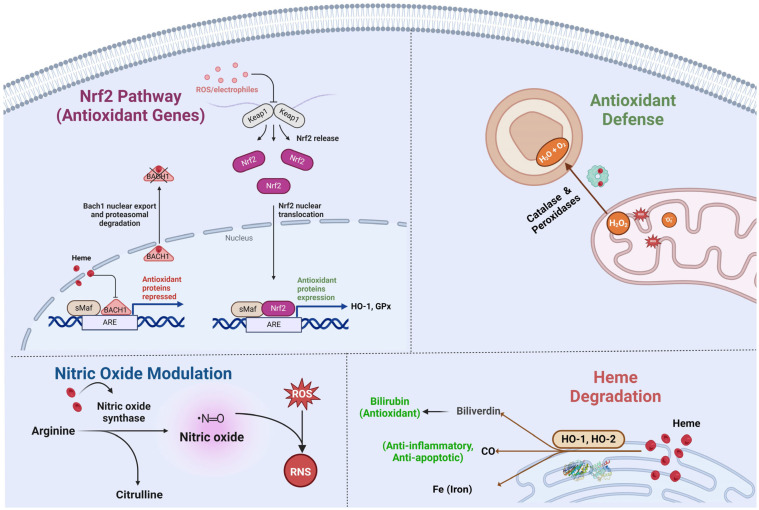
Cellular mechanisms of heme in neuroprotection and oxidative stress regulation. **Nrf2**-Nuclear factor erythroid 2–related factor 2. **ROS**-Reactive Oxygen Species. **sMaf**-Small Musculoaponeurotic Fibrosarcoma (Maf) protein. **Keap1**-Kelch-like ECH-associated protein 1. **ARE**-Antioxidant Response Element. **HO-1**-Heme Oxygenase-1. **HO-2**-Heme Oxygenase-2. **GPx**-Glutathione Peroxidase. **NO**-Nitric Oxide. **RNS**-Reactive Nitrogen Species. **CO**-Carbon Monoxide. **H₂O₂**-Hydrogen Peroxide. **Bach1**-BTB and CNC Homology 1. Created in BioRender. Soladogun, A. (2024). www.BioRender.com/q96j583 (accessed on 16 October 2024).

In addition to its direct antioxidant activity, heme also plays a pivotal role in cellular defense mechanisms against oxidative stress (Figure 4). This role is largely mediated through the activities of the heme degradation enzyme, heme oxygenase (HO-1 and HO-2) [144]. Heme oxygenase degrades heme into biliverdin, carbon monoxide (CO), and free iron [145]. These byproducts have significant neuroprotective properties [105,146].

Biliverdin is rapidly reduced to bilirubin by biliverdin reductase. Bilirubin is a potent lipophilic antioxidant that scavenges reactive oxygen species (ROS), including superoxide and hydrogen peroxide. Studies have shown that bilirubin can recycle between oxidized and reduced forms, allowing for prolonged antioxidant protection in neurons. This antioxidant cycling effectively neutralizes ROS, protecting cellular membranes and DNA from oxidative damage. The presence of biliverdin and bilirubin in neurons thus provides a local antioxidant defense that is particularly valuable in protecting against neurotoxic compounds and maintaining cellular integrity under stress conditions [62,147,148].

Carbon monoxide (CO), though toxic at high concentrations, acts as a signaling molecule at physiological levels. CO exerts its effects by activating soluble guanylate cyclase (sGC), which catalyzes the production of cyclic guanosine monophosphate (cGMP) from GTP. The increase in cGMP levels activates protein kinase G (PKG) and other downstream signaling cascades that support neuronal survival, reduce apoptosis, and enhance vascular health [149,150,151,152]. Through these pathways, CO can help modulate blood flow and oxygen supply to neurons, facilitating nutrient delivery and waste removal, which is particularly beneficial under ischemic conditions. Additionally, CO’s anti-inflammatory effects are mediated by its capacity to inhibit the release of pro-inflammatory cytokines from microglia and astrocytes, further protecting neurons from inflammation-induced damage [153].

Free iron, while potentially harmful due to its ability to catalyze the formation of harmful hydroxyl radicals through the Fenton reaction, is sequestered by ferritin, thus preventing iron-mediated oxidative damage [154,155,156]. This complex interplay of heme degradation products points to the sophisticated mechanisms through which heme contributes to neuronal health.

The induction of HO-1 is a critical adaptive response to oxidative stress in the brain. It is upregulated in response to various oxidative stimuli and plays a crucial role in the cellular defense against oxidative damage [157]. The neuroprotective effects of HO-1 have been demonstrated in various models of neurodegenerative diseases, including Alzheimer’s disease, Parkinson’s disease, and multiple sclerosis [158,159,160]. By mitigating oxidative stress and promoting cellular repair processes, HO-1 helps preserve neuronal integrity and function.

Additionally, heme influences the expression of other antioxidant defense genes through the activation of transcription factors such as nuclear factor erythroid 2–related factor 2 (Nrf2) [161,162]. Nrf2 is a master regulator of the antioxidant response, controlling the expression of numerous genes involved in oxidative stress defense. In addition to upregulating antioxidant genes such as HO-1, Nrf2 enhances the expression of genes involved in cellular repair and detoxification. For example, Nrf2 upregulates glutathione synthesis enzymes like glutamate–cysteine ligase (GCL) and glutathione reductase, which are crucial for the synthesis and recycling of glutathione, one of the most abundant intracellular antioxidants [163,164]. Glutathione is critical for maintaining cellular redox balance and detoxifying harmful substances. By enhancing glutathione levels, Nrf2 strengthens the cell’s intrinsic ability to neutralize ROS and repair oxidative damage.

Nrf2 also promotes the expression of detoxifying enzymes such as NAD(P)H quinone oxidoreductase 1 (NQO1). NQO1, for instance, reduces quinones to hydroquinones, thereby preventing redox cycling and reducing the formation of superoxide radicals, a major source of oxidative stress in neurons [165]. Nrf2 activation also impacts neuroinflammation by reducing the production of pro-inflammatory cytokines and upregulating genes that support anti-inflammatory responses [166]. This is achieved, in part, by the ability of Nrf2 to inhibit the nuclear factor kappa-light-chain-enhancer of activated B cells (NF-κB), a key regulator of inflammatory processes. By downregulating NF-κB activity, Nrf2 reduces the expression of cytokines such as IL-1β, TNF-α, and IL-6, which are implicated in the chronic neuroinflammation seen in diseases like Alzheimer’s and Parkinson’s [167]. This reduction in inflammation further supports neuronal health and resilience [168].

The interaction between heme and nitric oxide (NO) is another crucial aspect of its role in oxidative stress defense. NO, synthesized by nitric oxide synthase (NOS) enzymes, can react with ROS to form reactive nitrogen species (RNS), which can cause significant cellular damage. Heme-containing NOS enzymes regulate NO production, thereby modulating its levels and preventing the excessive formation of harmful RNS [67,169]. This regulation is vital in the nervous system, where NO acts as a neurotransmitter and signaling molecule involved in various physiological processes, including vasodilation, neurotransmission, and synaptic plasticity. Heme regulates the activity of other key antioxidant proteins. For example, it influences the activity of peroxiredoxins [170,171,172], proteins involved in reducing peroxides and maintaining redox homeostasis in cells. By regulating these proteins, heme ensures a balanced redox environment, crucial for neuronal health.

## 7. Heme Dysregulation and Neurological Disorders

### 7.1. Heme Metabolism and Alzheimer’s Disease

**Figure 5:**
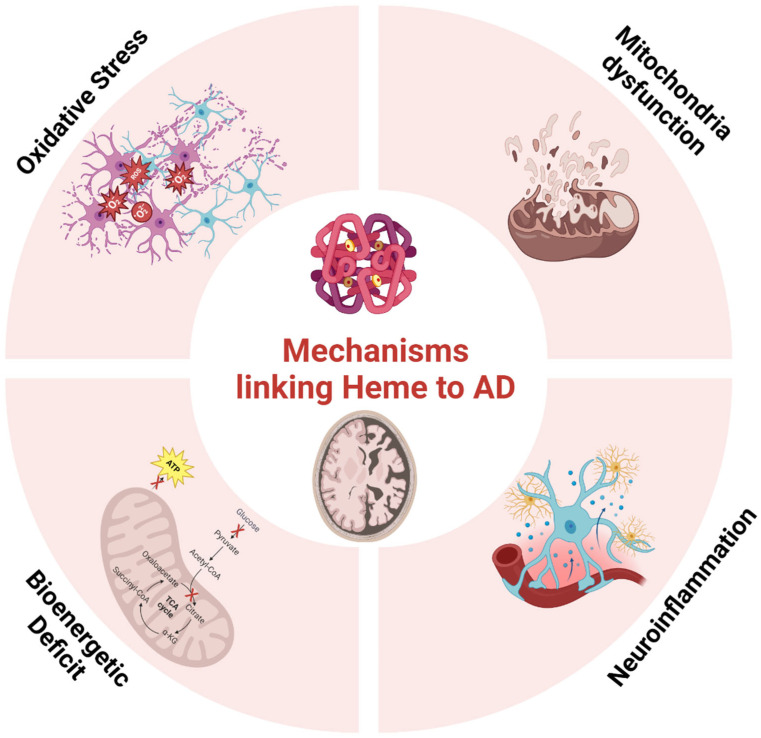
Mechanisms linking heme to Alzheimer’s disease. Created in BioRender. Soladogun, A. (2024). www.BioRender.com/g23a060 (accessed on 16 October 2024).

Alzheimer’s disease (AD) is a complex neurodegenerative disorder characterized by cognitive decline, memory loss, and extensive neuronal damage [173,174]. Emerging research has highlighted the critical role of heme metabolism in the pathogenesis of AD. Dysregulation of heme homeostasis can exacerbate oxidative stress, mitochondrial dysfunction, amyloid plaque formation, and neuroinflammation (Figure 5), all of which contribute to the progression of AD [175,176,177].

#### 7.1.1. Alteration of Heme Oxygenase-1 (HO-1) Activity

One of the primary mechanisms by which heme dysregulation contributes to AD is through the alteration of heme oxygenase-1 (HO-1) activity. HO-1 is an enzyme that degrades heme into biliverdin, carbon monoxide (CO), and free iron, each playing distinct roles in cellular metabolism and stress responses [105,144]. In AD patients, studies have demonstrated a significant reduction in HO-1 activity, which correlates with increased oxidative stress and neuronal damage [178,179]. This reduction in HO-1 activity can exacerbate the accumulation of free iron, leading to enhanced production of reactive oxygen species (ROS) and further neuronal injury [65,108].

HO-1 not only facilitates the degradation of heme but also produces byproducts with potent neuroprotective properties. Biliverdin is converted to bilirubin, which acts as a powerful antioxidant, scavenging free radicals and protecting cells from oxidative damage [144,150]. Carbon monoxide, while toxic at high concentrations, acts as a signaling molecule at physiological levels, exerting anti-inflammatory and anti-apoptotic effects that are beneficial in the context of neuroprotection [180,181]. Despite these protective roles, the dysregulation of HO-1 activity in AD disrupts these beneficial effects, leading to an environment conducive to oxidative stress and neuronal damage.

#### 7.1.2. Oxidative Stress and Mitochondrial Dysfunction

Oxidative stress is a hallmark of Alzheimer’s disease (AD) [182,183], and the dysregulation of heme metabolism exacerbates this condition. Heme is predominantly synthesized in the mitochondria, with four out of the eight steps of its biosynthesis occurring in this organelle [36]. Mitochondria are essential for cellular energy production via oxidative phosphorylation (OXPHOS), especially in the brain, which uses 20% of the oxygen despite comprising only 2% of body weight [184]. Neuronal functions, such as synaptic activities and brain information processes, depend almost entirely on ATP derived from OXPHOS. Impaired glucose metabolism, commonly seen in AD, further complicates energy production [185].

One of the primary mechanisms by which heme dysregulation contributes to oxidative stress in AD is through the release of iron. Free heme contains an iron atom that can be liberated when heme is degraded, especially under conditions of oxidative stress or elevated heme oxygenase-1 (HO-1) activity. This free iron is highly reactive and participates in the Fenton reaction, a chemical reaction in which ferrous iron (Fe^2+^) reacts with hydrogen peroxide (H_2_O_2_) to produce hydroxyl radicals (•OH), one of the most damaging types of reactive oxygen species (ROS):

Fe^2+^ + H_2_O_2_ → Fe^3+^ + OH^−^ + •OH

Hydroxyl radicals generated through the Fenton reaction are potent oxidizing agents that cause extensive lipid peroxidation, protein oxidation, and DNA damage in neurons [186]. In the context of AD, neurons are particularly vulnerable to oxidative stress due to their high metabolic activity and low regenerative capacity. Thus, the accumulation of free iron resulting from heme dysregulation creates a vicious cycle of oxidative damage, which accelerates neuronal death and contributes to the progression of AD [187,188].

**Figure 6:**
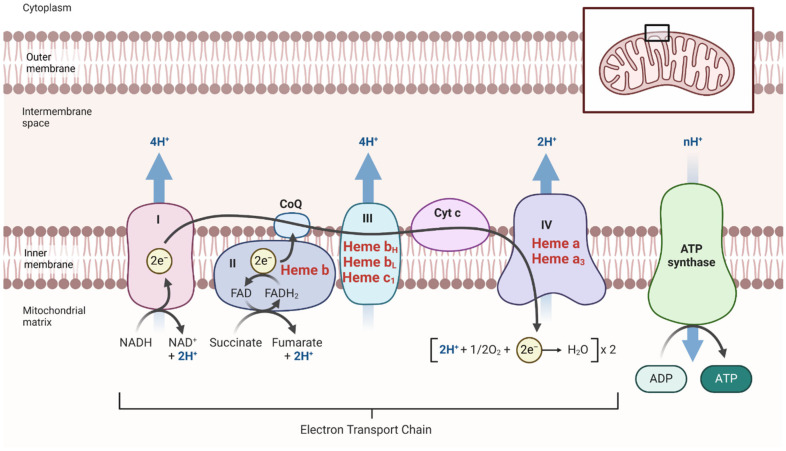
Heme is an essential component of OXPHOS complexes. **NADH**-Nicotinamide Adenine Dinucleotide (reduced form). **NAD^+^**-Nicotinamide Adenine Dinucleotide (oxidized form). **2H^+^**-Two protons. **CoQ**-Coenzyme Q (also known as ubiquinone). **FAD**-Flavin Adenine Dinucleotide. **FADH₂**-Flavin Adenine Dinucleotide (reduced form). **Cyt c**-Cytochrome c. **e^−^**-Electron. **O₂**-Molecular Oxygen. **H₂O**-Water. **ATP**-Adenosine Triphosphate. **ADP**-Adenosine Diphosphate. **nH^+^**-Multiple protons. **I**-Complex I (NADH: Ubiquinone oxidoreductase). **II**-Complex II (Succinate dehydrogenase complex). **III**-Complex III (Cytochrome bc_1_ complex). **IV**-Complex IV (Cytochrome c oxidase complex). Created in BioRender. Soladogun, A. (2024). www.BioRender.com/x49b585 (accessed on 16 October 2024).

Heme’s involvement in mitochondrial function is critical, as it is a component of several key enzymes and proteins within the electron transport chain (ETC) (Figure 6). The ETC enzymes, crucial for energy production, include several heme-containing components: cytochrome c (Cytc), and cytochrome c oxidase (COX). Cytochrome c is a peripheral membrane hemoprotein that serves as an electron carrier to COX and is involved in the regulation of oxidative phosphorylation (OXPHOS) [189]. Cytochrome c oxidase, which contains two heme subunits, couples the reduction of oxygen to water with the generation of a proton gradient across the mitochondrial membrane, serving as a key regulator of OXPHOS [190]. Specifically, heme-a serves as a crucial prosthetic group within cytochrome oxidase (COX) [191]. Increased levels of cerebral heme-a have been observed in Alzheimer’s disease (AD) patients, indicating a potential compensatory response to mitochondrial dysfunction [14].

The synthesis of heme is tightly linked to mitochondrial function. Heme biosynthesis has been shown to be coupled to electron transport for energy generation [192]. This creates a synergistic and feedback relationship where heme dysregulation leads to mitochondrial impairment, and mitochondrial deficiency can reduce heme synthesis and flux. This interdependence highlights the critical balance required for proper cellular function and energy homeostasis.

In AD, the dysregulation of heme metabolism can lead to mitochondrial dysfunction and increased oxidative stress [193]. The impaired function of mitochondrial enzymes, such as those in the ETC, results in reduced ATP production and increased production of reactive oxygen species (ROS), further contributing to neuronal damage [194,195]. The role of oxidative stress in AD is multifaceted, impacting several cellular components and processes. Mitochondrial dysfunction, a key contributor to oxidative stress, generates excessive ROS, damaging mitochondrial DNA and disrupting ATP production, leading to energy deficits in neurons. The interplay between mitochondrial dysfunction and oxidative stress creates a vicious cycle, exacerbating neuronal damage and contributing to the progression of AD [196,197].

In summary, heme is required for proper mitochondrial energy generation and function, and mitochondria are crucial for heme biosynthesis. This interdependence underscores the importance of maintaining heme and mitochondrial homeostasis to prevent the exacerbation of oxidative stress and neuronal damage in AD.

#### 7.1.3. Heme and Amyloid Plaque Formation

The accumulation of amyloid-beta (Aβ) peptides, which form the core of amyloid plaques, has been linked to disturbances in iron homeostasis. Heme binds to Aβ peptides, affecting their aggregation and contributing to plaque formation [198,199]. This binding of heme to Aβ is significant as it alters the metabolism of both heme and Aβ, exacerbating disease pathology [200]. Heme oxygenase activity influences the degradation of Aβ, as iron released from heme catabolism can influence Aβ aggregation and plaque formation. Iron can directly interact with Aβ peptides, promoting their aggregation and stabilizing plaque formation. Research indicates that iron binds to histidine residues in the Aβ sequence, facilitating peptide aggregation into insoluble fibrils [201]. This aggregation is significant because Aβ plaques are not only a hallmark of AD but also a source of additional oxidative stress. Plaques are associated with localized microenvironments that generate ROS, further increasing neuronal damage [202].

Research has demonstrated that low cerebrospinal fluid levels of hemopexin, a heme-binding protein that detoxifies free heme, are associated with increased AD pathology. Hemopexin helps maintain iron homeostasis and exhibits antioxidant properties. Its deficiency can lead to enhanced oxidative stress and amyloid pathology in AD [203]. This relationship underscores the importance of heme regulation in preventing Aβ aggregation and the subsequent formation of amyloid plaques.

The deposition of amyloid plaques is not only a marker of AD but also a driver of neurotoxicity. Aβ peptides can induce oxidative stress and inflammatory responses, further damaging neurons and synapses. The presence of iron in amyloid plaques exacerbates this damage by promoting the formation of reactive oxygen species (ROS), leading to a cycle of oxidative damage and neurodegeneration [204,205]. Therefore, managing heme metabolism and iron homeostasis is crucial for mitigating amyloid plaque formation and its deleterious effects.

#### 7.1.4. Neuroinflammation and Heme Dysregulation

Neuroinflammation is a hallmark of Alzheimer’s disease (AD) pathogenesis, and heme metabolism emerges as a crucial modulator of this inflammatory response. Heme and its degradation products, particularly carbon monoxide (CO) and biliverdin/bilirubin, exert anti-inflammatory effects [159]. However, dysregulated heme metabolism in AD can lead to exacerbated neuroinflammation, contributing to neuronal damage and disease progression.

Studies have revealed peripheral immune dysregulation in AD patients, characterized by altered levels of heme oxygenase-1 (HO-1), the rate-limiting enzyme in heme degradation, and other inflammatory markers [206,207]. These findings underscore the systemic nature of inflammation in AD and the potential involvement of heme metabolism in this process.

Neuroinflammation in AD involves the activation of glial cells, including microglia and astrocytes. Activated glia releases pro-inflammatory cytokines and chemokines, amplifying the inflammatory cascade and causing neuronal damage [208,209]. HO-1, by generating anti-inflammatory molecules like CO and biliverdin/bilirubin, typically mitigates this inflammatory response and protects neurons [105,210]. However, the dysregulation of HO-1 in AD disrupts this delicate balance, fostering a chronic inflammatory milieu that fuels disease progression [211].

The chronic inflammatory environment not only directly damages neurons but also disrupts synaptic function and plasticity. Pro-inflammatory cytokines can interfere with neurotransmitter signaling and synaptic maintenance, further compromising cognitive abilities [212]. The interplay between neuroinflammation and oxidative stress, another key factor in AD pathology, establishes a vicious cycle that accelerates neurodegeneration [183].

Targeting heme metabolism to restore the balance between pro- and anti-inflammatory signals holds promise for therapeutic intervention in AD. By modulating HO-1 activity and promoting the production of anti-inflammatory molecules, it may be possible to dampen neuroinflammation and protect neurons from further damage [213].

### 7.2. Parkinson’s Disease (PD) and the Role of Heme Imbalance

Parkinson’s disease (PD) is a progressive neurodegenerative disorder characterized by motor dysfunction, including tremors, rigidity, and bradykinesia. While the exact cause of PD remains elusive, mounting evidence suggests that an imbalance in heme metabolism plays a significant role in its pathogenesis [214]. Several mechanisms have been proposed to explain the link between heme imbalance and PD; these include the following mechanisms described below.

#### 7.2.1. Accumulation of Heme Iron in the Substantia Nigra

The substantia nigra, a brain region rich in dopaminergic neurons, is particularly vulnerable to degeneration in PD. Studies have demonstrated an accumulation of iron, a component of heme, in the substantia nigra of PD patients [215,216]. This accumulation contributes to oxidative stress and neurodegeneration. Iron-related MRI studies have shown increased iron levels in the substantia nigra of PD patients, correlating with disease severity [217]. Quantitative susceptibility mapping (QSM) has further confirmed increased iron deposition in the substantia nigra, particularly in advanced stages of PD [218].

Iron accumulation in the substantia nigra plays a pivotal role in the pathogenesis of PD. Excess iron can catalyze the formation of highly reactive hydroxyl radicals through the Fenton reaction, leading to oxidative damage of lipids, proteins, and nucleic acids. Postmortem studies have revealed significantly higher levels of iron in the substantia nigra of PD patients compared to controls [9,219]. This iron accumulation is thought to contribute to the selective vulnerability of dopaminergic neurons in PD.

#### 7.2.2. Heme Interaction with α-Synuclein

α-Synuclein is a synaptic protein that is abundant in the brain and plays a critical role in maintaining synaptic vesicle dynamics [220]. However, in PD, α-synuclein undergoes misfolding and aggregation, forming insoluble fibrils that are deposited as Lewy bodies [221]. Research has shown that heme can bind to α-synuclein, influencing its structural properties and promoting its aggregation [222]. The interaction between heme and α-synuclein promotes protein aggregation and can also contribute to the neurotoxic effects observed in PD. Aggregated α-synuclein can disrupt various cellular functions, including mitochondrial activity, synaptic transmission, and protein degradation pathways [223]. The presence of heme-bound α-synuclein aggregates has been associated with increased oxidative stress, mitochondrial dysfunction, and impaired autophagy, all of which are key features of PD pathology.

#### 7.2.3. Heme-Related Pathways and Neuroinflammation

Heme dysregulation significantly influences neuroinflammatory pathways in Parkinson’s disease (PD. The expression and activity of HO-1, which are crucial for maintaining cellular antioxidant responses and modulating inflammatory processes, are altered in PD. Studies have shown that in PD patients, the expression of HO-1 in the substantia nigra is disrupted. Some research indicates a decrease in HO-1 expression in this brain region [224].

This dysregulation of HO-1 can significantly impact the cellular antioxidant response, as HO-1 is responsible for breaking down heme into biliverdin, CO, and free iron, which are critical for various cellular functions. Increased iron levels resulting from impaired heme degradation can activate microglia, the resident immune cells of the brain. Activated microglia releases pro-inflammatory cytokines and other inflammatory mediators, further exacerbating neuroinflammation and contributing to neuronal damage [225].

The alterations in HO-1 activity disrupt the delicate balance of heme metabolism, leading to increased oxidative stress and inflammation, which are key contributors to the pathophysiology of PD. The resulting neuroinflammatory environment can enhance neuronal vulnerability to degeneration, particularly in dopaminergic neurons of the substantia nigra, thus driving the progression of PD [226,227].

### 7.3. Heme’s Involvement in Traumatic Brain Injuries (TBI) and Recovery

Traumatic brain injury (TBI) is a significant cause of morbidity and mortality worldwide, characterized by complex pathophysiological processes involving primary and secondary brain damage. The secondary injury mechanisms, which include oxidative stress, inflammation, and cell death, often exacerbate the initial damage. Emerging research has highlighted the crucial role of heme metabolism in the pathogenesis and recovery of TBI. Heme, an iron-containing molecule essential for various biological functions, can influence the outcome of TBI through its involvement in oxidative stress response, inflammation, and cellular repair mechanisms.

#### 7.3.1. Role of Hemoglobin in Traumatic Brain Injury

In traumatic or hemorrhagic brain injury, the breakdown of blood vessels often leads to the release of cell-free hemoglobin into the brain environment. Hemoglobin, primarily a transporter of oxygen within red blood cells, becomes a source of free heme once it enters the extracellular space [228]. This release initiates a cascade of harmful effects, as hemoglobin breaks down and releases heme, an iron-containing molecule, which subsequently contributes to oxidative stress, iron toxicity, and inflammation in brain tissue.

Following brain hemorrhage, free hemoglobin undergoes oxidation, converting to reactive forms such as methemoglobin and ferryl hemoglobin. Methemoglobin, an oxidized form of hemoglobin, is prone to further breakdown into heme and iron, both of which are toxic in the unbound state [229,230]. Ferryl hemoglobin is an even more reactive form, capable of generating reactive oxygen species (ROS) directly, leading to lipid peroxidation, protein modification, and DNA damage within neurons and glial cells. The presence of these oxidized hemoglobin species in the brain has been linked to exacerbated oxidative damage, contributing to neuronal death and the progression of secondary brain injury [231,232].

#### 7.3.2. Heme’s Dual Role in TBI-Associated Oxidative Damage

Heme plays a dual role in TBI, acting as both a damaging agent and a potential therapeutic target. Immediately following TBI, the disruption of the blood–brain barrier and cellular injury leads to the release of hemoglobin and other hemoproteins. This free heme, in excess, can act as a pro-oxidant, catalyzing the formation of reactive oxygen species (ROS) and exacerbating oxidative stress, a major contributor to secondary injury after [233]. Moreover, heme can directly activate inflammatory pathways, triggering the release of pro-inflammatory cytokines and contributing to neuroinflammation, further exacerbating neuronal damage [234,235].

However, heme also has a protective role in TBI recovery. Heme oxygenase (HO), the enzyme responsible for heme degradation, converts heme into biliverdin, carbon monoxide (CO), and free iron. Biliverdin and CO have potent antioxidant and anti-inflammatory properties, offering protection against the detrimental effects of oxidative stress and inflammation [159,236]. Additionally, CO has been shown to modulate vascular tone, potentially improving cerebral blood flow and oxygen delivery to injured brain regions [237,238].

HO-2, on the other hand, is constitutively expressed in the brain and is predominantly localized in neurons. Although less studied than HO-1, HO-2 has also been shown to have neuroprotective effects after TBI. HO-2 knockout mice exhibit increased neuronal cell death, impaired motor recovery, and decreased ability to reduce oxidative stress after TBI, suggesting that HO-2 is essential for neuronal survival and repair [239].

#### 7.3.3. Heme and Neuroinflammation in TBI

Neuroinflammation is a critical secondary injury mechanism in TBI, and heme metabolism significantly influences this process. The release of free heme can activate microglia and astrocytes, the primary immune cells in the brain, leading to the production of pro-inflammatory cytokines and chemokines [240]. This inflammatory response, while initially protective, can become chronic and contribute to ongoing neuronal damage and dysfunction.

HO-1 induction plays in important role in modulating neuroinflammation. On one hand, it degrades free heme, reducing its pro-inflammatory potential. On the other hand, the byproducts of heme degradation, particularly CO, exert significant anti-inflammatory effects in TBI recovery by modulating key signaling cascades, including the p38 mitogen-activated protein kinase (p38 MAPK) pathway. In response to injury, CO activates p38 MAPK, which subsequently leads to the phosphorylation of downstream transcription factors such as ATF-2 and NF-κB. This activation reduces the production of pro-inflammatory cytokines, including tumor necrosis factor-alpha (TNF-α) and interleukin-1 beta (IL-1β), thereby decreasing inflammation in the injured brain [241].

CO’s activation of the p38 MAPK pathway also influences macrophage and microglial polarization. CO promotes a shift from the pro-inflammatory M1 phenotype to the anti-inflammatory M2 phenotype in these immune cells, reducing inflammation and promoting tissue repair [241]. This shift enhances the release of anti-inflammatory cytokines such as IL-10 and TGF-β, creating a neuroprotective environment conducive to neuronal survival and regeneration. Biliverdin and bilirubin have been shown to suppress microglial activation and reduce the production of inflammatory mediators [242]. Therefore, the HO-1 pathway serves as a crucial regulatory mechanism in controlling neuroinflammation following TBI.

#### 7.3.4. Heme and Cellular Repair in TBI

In addition to its roles in oxidative stress and inflammation, heme metabolism also influences cellular repair mechanisms in TBI. The byproducts of heme degradation, particularly CO, have been implicated in promoting angiogenesis and neurogenesis, which are essential for brain repair and recovery. CO can enhance the expression of vascular endothelial growth factor (VEGF), a key mediator of angiogenesis, and promote the proliferation and differentiation of neural progenitor cells [243,244].

Furthermore, the heme oxygenase (HO)/carbon monoxide (CO) and heme-containing nitric oxide synthase (NOS)/nitric oxide (NO) axis is critical for cellular repair following traumatic brain injury (TBI) [244]. Induced by TBI, HO degrades excess heme from damaged cells, producing CO. CO subsequently activates NOS, increasing NO production. Both CO and NO exhibit anti-inflammatory and vasodilatory effects, which are vital for minimizing secondary injury and promoting tissue repair. Additionally, NO plays a significant role in stimulating angiogenesis, neurogenesis, and synaptogenesis, primarily through the vascular endothelial growth factor (VEGF) signaling pathway. NO can enhance the expression of VEGF, a growth factor essential for angiogenesis and neurogenesis, by activating hypoxia-inducible factor-1 alpha (HIF-1α) under hypoxic or inflammatory conditions [245]. HIF-1α induces VEGF expression, which, in turn, binds to VEGF receptors on neural progenitor cells, activating downstream signaling pathways that promote cell survival, proliferation, and differentiation [246]. These effects contribute to the protection and regeneration of neurons following TBI.

### 7.4. Diseases Caused by Mutations in Heme Transporters

Mutations in Feline Leukemia Virus Subgroup C Receptor 1 (*FLVCR1*), a critical heme transporter, lead to impaired heme export, disrupting heme homeostasis and resulting in neurodegenerative diseases. *FLVCR1* ensures intracellular heme regulation by transporting excess heme out of cells, preventing toxic accumulation. Mutations in this transporter impair heme efflux, leading to oxidative stress, mitochondrial dysfunction, and neurodegeneration.

#### 7.4.1. *FLVCR1* Mutations and PCARP Syndrome

One of the most significant clinical manifestations of *FLVCR1* mutations is Posterior Column Ataxia and Retinitis Pigmentosa (PCARP). PCARP is a rare autosomal recessive disorder that presents proprioceptive sensory deficits, progressive ataxia, and retinal degeneration, ultimately leading to vision loss and sensory neuropathy [247].

Studies using gene sequencing techniques have identified several pathogenic mutations in *FLVCR1*—including missense and frameshift mutations—that impair the transporter’s ability to regulate heme export effectively. These mutations result in toxic intracellular heme buildup, particularly affecting the posterior columns of the spinal cord and retinal neurons, areas highly reliant on precise heme regulation for normal functioning [248,249].

#### 7.4.2. Molecular Mechanisms of Neurodegeneration

FLVCR1 has two primary isoforms with distinct roles in cellular heme regulation. FLVCR1a, located on the plasma membrane, exports free heme to protect cells from oxidative damage, while FLVCR1b, located on mitochondrial membranes, facilitates the export of newly synthesized heme. The impairment of either isoform causes mitochondrial dysfunction and triggers reactive oxygen species (ROS) production, accelerating neuronal degeneration [250,251].

Mouse models and patient studies have shown that the highest expression levels of FLVCR1 are found in the retina and spinal cord, explaining the tissue-specific degeneration seen in PCARP. Patients with *FLVCR1* mutations exhibit sensory neuropathy, proprioceptive deficits, and visual impairment, as the dysfunctional transporter compromises the health of neurons critical to proprioception and retinal function [247,248].

Understanding the role of FLVCR1 in heme metabolism opens avenues for therapeutic interventions targeting heme export pathways. Approaches that mitigate heme toxicity or enhance residual FLVCR1 function could delay the progression of neurodegeneration in PCARP patients. Additionally, monitoring heme levels in individuals with known *FLVCR1* mutations may offer diagnostic insights for early intervention in sensory and visual neuropathies.

## 8. Therapeutic Potential and Future Directions: Harnessing the Heme Pathway for Neurological Interventions

The multifaceted roles of heme in the nervous system, as outlined in this review, present compelling opportunities for therapeutic intervention in various neurological disorders. By targeting heme metabolism, its downstream signaling pathways, and its interactions with key cellular components, researchers aim to develop novel strategies for neuroprotection, disease modification, and even diagnosis.

### 8.1. Heme-Targeted Therapies for Neurodegenerative Diseases

Given the detrimental effects of heme dysregulation in neurodegenerative diseases such as Alzheimer’s and Parkinson’s, strategies to restore heme homeostasis or modulate its downstream effects are actively being pursued.

HO, the enzyme that degrades heme, is a primary target for therapeutic intervention. Upregulating HO-1 activity has shown promise in preclinical models of neurodegenerative diseases. For instance, in Alzheimer’s disease, HO-1 induction has been shown to reduce amyloid-beta (Aβ) aggregation and associated neurotoxicity through the production of biliverdin/bilirubin and ferritin, which have antioxidant and iron-sequestering properties, respectively [236]. This reduction in the Aβ burden can help alleviate neuroinflammation and synaptic dysfunction, key contributors to cognitive decline in AD. In Parkinson’s disease, enhancing HO-1 activity can attenuate dopaminergic neuronal loss and improve motor function by reducing oxidative stress and inflammation in the substantia nigra [105].

Pharmacological agents that induce HO-1, such as hemin and cobalt protoporphyrin (CoPP), are being investigated for their potential to delay disease progression. For example, hemin has shown promise in preclinical PD models by protecting dopaminergic neurons and improving motor deficits. However, challenges remain in optimizing drug delivery and ensuring long-term safety and efficacy [252].

### 8.2. Carbon Monoxide (CO)-Releasing Molecules (CORMs)

CORMs, which deliver controlled amounts of CO, have emerged as a novel therapeutic approach [253]. CO, a product of heme degradation by HO, has potent anti-inflammatory, anti-apoptotic, and cytoprotective effects. Preclinical studies have shown that CORMs can mitigate neuronal damage and improve functional outcomes in models of stroke, TBI, and neurodegenerative diseases [54]. For example, in models of Parkinson’s disease, low dose CO has been shown to reduce dopaminergic neuron loss and alleviate motor symptoms [254]. The therapeutic potential of CORMs lies in their ability to mimic the endogenous protective effects of CO without the potential toxicity associated with high concentrations of inhaled CO. However, the development of clinically viable CORMs requires careful consideration of dosage, release kinetics, and potential off-target effects.

### 8.3. Targeting Heme Transport and Trafficking

Modulating heme transport and trafficking within the nervous system represents another potential therapeutic strategy. Heme transport and homeostasis are tightly regulated processes, and disruptions in these processes can contribute to neurodegenerative diseases. For example, in Alzheimer’s disease, impaired heme transport has been linked to mitochondrial dysfunction and oxidative stress [255]. By targeting proteins involved in heme uptake, export, and intracellular trafficking, it may be possible to restore heme balance and reduce its detrimental effects in the brain.

### 8.4. Hemoglobin Scavenging and Detoxification

Haptoglobin serves as a key scavenger protein that binds to free hemoglobin, thereby limiting its toxicity in the brain following hemorrhagic injury. Haptoglobin forms stable complexes with free hemoglobin, reducing the release of free heme and decreasing hemoglobin’s oxidative capacity. When haptoglobin binds hemoglobin, it prevents the breakdown of hemoglobin into heme and iron, thereby curbing the potential for oxidative damage mediated by free iron and heme [256,257].

The haptoglobin–hemoglobin complex is recognized by the CD163 receptor on macrophages and microglia, facilitating the clearance of hemoglobin from the extracellular space and its safe degradation within phagocytic cells [258,259]. This pathway reduces hemoglobin-induced oxidative stress also lowers inflammation by minimizing the exposure of neural tissues to free heme, a pro-oxidant and inflammatory agent. Research has shown that, in models of traumatic brain injury (TBI) and hemorrhagic stroke, haptoglobin expression is upregulated as a compensatory mechanism to mitigate hemoglobin toxicity. In these models, higher levels of haptoglobin correlate with reduced inflammation and oxidative damage, supporting neuronal survival and enhancing overall recovery [260,261]. Targeting haptoglobin pathways or enhancing its expression in the brain could therefore offer a promising approach to managing the effects of hemoglobin release and limiting secondary damage in brain injuries.

### 8.5. Heme Detoxification and Iron Chelation

Hemopexin (HPX) and Alpha-1-microglobulin (A1M) are heme binding proteins that detoxify free heme and maintain iron homeostasis, playing a crucial role in mitigating the deleterious effects of heme dysregulation in neurodegenerative diseases. Preclinical studies have shown that increasing hemopexin levels can reduce oxidative stress and inflammation in models of brain injury and neurodegeneration [262].

Alpha-1-microglobulin (A1M) is a multifunctional scavenger protein with a significant role in binding free heme and neutralizing reactive oxygen species (ROS), providing protection against oxidative damage in various tissues, including the brain. A1M acts as an antioxidant by binding to free heme, thereby limiting its oxidative capacity and preventing cellular damage [263]. Its heme-binding ability is coupled with a unique reductase activity, allowing A1M to detoxify free radicals and neutralize ROS, which are major contributors to neurodegenerative processes and brain injury [264].

Research has demonstrated that A1M levels are altered in several neurological disorders, particularly in conditions involving hemorrhagic injury and neurodegeneration, such as traumatic brain injury (TBI) and Alzheimer’s disease [265]. In these contexts, A1M expression is often upregulated in response to increased oxidative stress and inflammation, suggesting a protective role. Studies indicate that enhanced A1M expression in brain tissues exposed to heme and ROS contributes to the reduction in oxidative damage, ultimately supporting neuronal survival and repair [266,267].

Another promising approach is the use of iron chelators to reduce the levels of free iron in the brain, as iron overload can exacerbate oxidative damage and neuroinflammation. Deferoxamine, an FDA-approved iron chelator, has shown potential in reducing iron-mediated oxidative damage and improving cognitive function in animal models of AD [268].

Additionally, antioxidant therapies that target the downstream effects of heme dysregulation are being explored. Compounds such as N-acetylcysteine, alpha-lipoic acid, and resveratrol have demonstrated antioxidant and neuroprotective effects in preclinical studies, and their potential benefits in AD are being investigated in clinical trials [269,270]. These therapies aim to neutralize the excess reactive oxygen species generated by heme dysregulation, thereby protecting neurons from oxidative damage and reducing neuroinflammation.

## 9. Diagnostic Potential of Heme-Related Biomarkers in Neurodegeneration

The involvement of heme metabolism in neurodegenerative diseases also presents opportunities for developing diagnostic biomarkers. Heme and its metabolites, along with proteins involved in heme metabolism, can serve as indicators of disease presence and progression.

### 9.1. Heme Metabolic Enzymes

Heme oxygenase-1 (HO-1) is an inducible enzyme that degrades heme into biliverdin, carbon monoxide (CO), and free iron. Elevated levels of HO-1 in cerebrospinal fluid (CSF) and brain tissue have been linked to neurodegenerative diseases, making it a potential biomarker for disease activity and therapeutic efficacy [271,272]. Research has demonstrated altered HO-1 expression in the brains of patients with Alzheimer’s disease (AD) and Parkinson’s disease (PD), correlating with disease severity and progression [273].

Additionally, biliverdin reductase-A (BLVRA) plays a crucial role in heme metabolism by converting biliverdin to bilirubin, a molecule with significant antioxidant properties. Altered levels and activity of BLVRA have been observed in the brains of individuals with Alzheimer’s disease and mild cognitive impairment. These changes in BLVRA expression suggest a disruption in heme metabolism, further emphasizing its role in the pathophysiology of neurodegenerative diseases and its potential as a diagnostic biomarker [274].

### 9.2. Cerebrospinal Fluid Heme and Metabolites

Quantifying heme and its degradation products, such as biliverdin and bilirubin, in CSF can aid in the diagnosis and monitoring of neurodegenerative diseases. Altered levels of these metabolites reflect the underlying dysregulation of heme metabolism and oxidative stress. Studies have shown that patients with AD exhibit increased levels of free heme and decreased levels of hemopexin in CSF, suggesting their potential as biomarkers for early diagnosis [203].

### 9.3. Iron and Ferritin Levels

Iron accumulation is a hallmark of several neurodegenerative diseases, and measuring iron and ferritin levels in CSF and blood can provide valuable diagnostic information. Elevated iron levels have been linked to disease progression in AD and PD, and iron chelation therapy’s efficacy can be monitored through these biomarkers [275,276].

### 9.4. Proteomic and Genomic Approaches

Advances in proteomics and genomics have enabled the identification of novel biomarkers related to heme metabolism. High-throughput techniques can identify changes in the expression of genes and proteins involved in heme synthesis, degradation, and transport. These approaches can uncover new biomarkers that offer greater specificity and sensitivity for diagnosing neurodegenerative diseases [277,278].

In summary, the potential of heme as a diagnostic biomarker represents a promising avenue for addressing the challenges posed by neurodegenerative diseases. Biomarkers related to heme metabolism can enhance early diagnosis and monitoring of disease progression.

## 10. Conclusions

This review highlights the intricate roles of heme in neural physiology and disease, emphasizing its importance in neurotransmission, oxidative stress regulation, and neurodevelopment. Beyond its classical roles in oxygen transport and electron transfer, heme functions as a modulator of ion channels, neurotransmitter synthesis, and synaptic plasticity. However, disruptions in heme homeostasis—due to mutations in heme transporters such as *FLVCR1* or impaired heme–regulatory protein interactions—can result in neurodegenerative diseases, including Posterior Column Ataxia and Retinitis Pigmentosa (PCARP), Alzheimer’s disease, and Parkinson’s disease.

We also explored the mechanisms by which heme activates the Nrf2 pathway through interactions with Bach1, ensuring antioxidant defense and cellular adaptation to oxidative stress. These mechanisms illustrate the dual nature of heme as both a necessary biological molecule and a potential source of toxicity if dysregulated. The regulation of Nrf2 by both Bach1 and Keap1 highlights the complexity of redox control systems that safeguard cellular health under stress.

Given the role of heme dysregulation in several neurodegenerative conditions, future research should focus on understanding heme signaling and developing targeted therapies. Modulating the heme–Nrf2 axis and restoring heme transporter function may offer therapeutic potential in diseases with oxidative and neuroinflammatory components. Further investigation into the biomarkers of heme metabolism could also pave the way for early diagnostic tools, improving patient outcomes.

In summary, the multifaceted involvement of heme in neural biology underscores its significance beyond traditional hematological contexts. Recognizing heme as a central player in the nervous system not only broadens our understanding of neurobiology but also provides a foundation for innovative therapeutic approaches targeting heme pathways in neurodegenerative diseases.

## Data Availability

No new data were generated or analyzed in this study, and therefore, data sharing is not applicable.
